# Low-frequency vibratory exercise reduces the risk of bone fracture more than walking: a randomized controlled trial

**DOI:** 10.1186/1471-2474-7-92

**Published:** 2006-11-30

**Authors:** Narcís Gusi, Armando Raimundo, Alejo Leal

**Affiliations:** 1 Faculty of Sports Sciences, University of Extremadura, Cáceres, Spain; 2 Department of Health and Welfare, University of Évora, Évora, Portugal; 3 Unite of Traumathology, Hospital of Cáceres, Cáceres, Spain

## Abstract

**Background:**

Whole-body vibration (WBV) is a new type of exercise that has been increasingly tested for the ability to prevent bone fractures and osteoporosis in frail people. There are two currently marketed vibrating plates: a) the whole plate oscillates up and down; b) reciprocating vertical displacements on the left and right side of a fulcrum, increasing the lateral accelerations. A few studies have shown recently the effectiveness of the up-and-down plate for increasing Bone Mineral Density (BMD) and balance; but the effectiveness of the reciprocating plate technique remains mainly unknown. The aim was to compare the effects of WBV using a reciprocating platform at frequencies lower than 20 Hz and a walking-based exercise programme on BMD and balance in post-menopausal women.

**Methods:**

Twenty-eight physically untrained post-menopausal women were assigned at random to a WBV group or a Walking group. Both experimental programmes consisted of 3 sessions per week for 8 months. Each vibratory session included 6 bouts of 1 min (12.6 Hz in frequency and 3 cm in amplitude with 60° of knee flexion) with 1 min rest between bouts. Each walking session was 55 minutes of walking and 5 minutes of stretching. Hip and lumbar BMD (g·cm^-2^) were measured using dual-energy X-ray absorptiometry and balance was assessed by the blind flamingo test. ANOVA for repeated measurements was adjusted by baseline data, weight and age.

**Results:**

After 8 months, BMD at the femoral neck in the WBV group was increased by 4.3% (*P *= 0.011) compared to the Walking group. In contrast, the BMD at the lumbar spine was unaltered in both groups. Balance was improved in the WBV group (29%) but not in the Walking group.

**Conclusion:**

The 8-month course of vibratory exercise using a reciprocating plate is feasible and is more effective than walking to improve two major determinants of bone fractures: hip BMD and balance.

## Background

Bone fracture is among the commonest and most expensive health problems in the population, particularly in postmenopausal women [1]. The major determinants of bone fractures are falls, bone fragility, loss of balance and decrease of lower limb strength[2,3]. Physical exercise is considered as an effective strategy, frequently recommended in general practice, for the prevention and management of postmenopausal osteoporosis[4,5]. Aerobics, weight bearing and resistance exercises were all effective increasing bone mass density[6]. However, arduous bone stress induced by vigorous weight-bearing activities can increase the risk of injuries, particularly in the elderly[7]. Therefore, alternative strategies with a lower risk of injury have been sought and usually included in the medical advice, such as walking programmes[8]. Walking and moderate-intensity aerobic exercise programmes have been shown to reduce bone loss although they did not increase significantly bone mineral density (BMD) compared to controls in the first few years of menopause[4] and they showed limited effects on bone in postmenopausal women[9].

Vibration could be a viable alternative in frail people[10,11]. Whole-body vibration (WBV) is a new type of exercise that has been increasingly tested for the ability to prevent bone fractures and osteoporosis[3,12-15]. Recent studies of WBV have shown a positive effect of controlled WBV on gait, body balance and motor capacity[16]. However, the treatment has to follow specific safety guidelines[17] to prevent exercise-related injuries (back pain, muscular discomfort, etc.), such as limiting the exposure to vibration to a maximum of 10 minutes and maintaining the posture of the participant in a semi-squat stance with knees flexed, with active involvement of the leg muscles to reduce the transmission of vibration to the head.

The currently marketed devices that deliver sinusoidal vibration to the whole body use two different types of vibrating plates[17]: a) the whole plate oscillates up and down; b) reciprocating vertical displacements on the left and right side of a fulcrum, increasing the lateral accelerations. A few studies have shown recently that there is scarce evidence of the effectiveness of the up-and-down plate for increasing BMD in experimental animals[13] or humans[15]; but the literature is largely lacking studies of the effect of the reciprocating plate technique.

Currently available WBV exercise devices deliver vibrations at frequencies of 15–60 Hz. Investigators usually administered frequencies at 15–35 Hz to obtain the maximum transmissibility of the mechanical stimulus produced by the vibrating plate[18]. On the other hand, since the resonance frequency for the WBV is in the range of 5–10 Hz [19], the frequencies lower than 20 Hz has been usually avoided. However, some recent studies have included in their protocols frequencies at 10–15 Hz to allow for gentle adaptation in frail populations (nursing home residents, elder, rehabilitation programs, etc.)[16,20]. But, to our knowledge, none of them have reported the specific effect of these low frequencies on bone mass. This knowledge could specially contribute to make decisions on the WBV programs to frail people. However, prior to administer these WBV programs in frail people, these programs have to be tested in healthy population.

The purpose of the current study is to determine whether 8 months of WBV exercise at 12.6 Hz using a reciprocating plate is more effective than walking for improving BMD and balance in healthy postmenopausal women.

## Methods

### Subjects and study design

**Figure 1:**
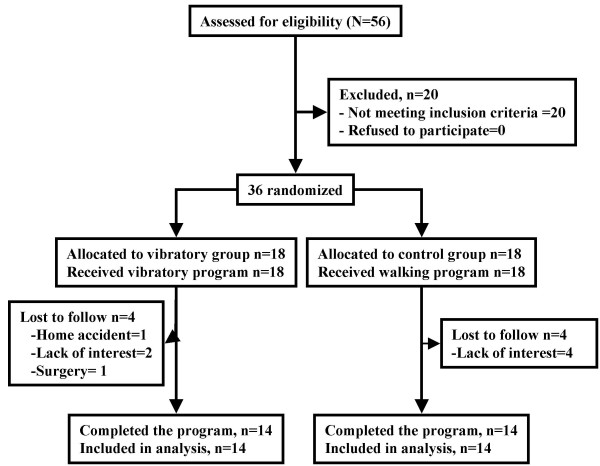
Flow-chart of participants throughout trial.

Figure 1 shows that 36 postmenopausal women recruited through advertisements in local newspapers volunteered to participate in the study; however, only the 28 who completed the trial are included in the analysis. Informed consent was obtained from all qualified volunteers. The inclusion criteria were: at least 5 years from the last menstruation; adequate nutritional status according to WHO norms (as determined by questionnaire); non-smoker; consumption of no more than four alcoholic beverages per week; the ability to follow the protocol; free from disease or medication known to affect bone metabolism or muscle strength. A questionnaire designed to gather information about current and previous dietary factors, including intake of calcium and vitamin D, was administered. At baseline, serum osteocalcin and urinary deoxypyridinoline crosslinks and creatinine were determined as markers of bone formation and resorption, respectively[21].

Exclusion criteria were: acute hernia; thrombosis; any pharmacologic intervention for osteopenia within the previous 6 months; any history of severe musculoskeletal problems; engaged in high-impact activity at least twice a week (any weight-bearing activity or exercise more intense than brisk walking).

All subjects were assigned at random to one of the study groups. A total of 14 women trained for 8 months on a vibrating plate via reciprocation (the WBV group). The other 14 women participated in a walking activity (the Walking group). The WBV programme consisted of 96 training sessions within a period of 32 weeks. The frequency of training was three times a week, with at least 1 day of rest between sessions in both experimental groups.

This study was approved by the University's Bioethics Committee according to the Helsinki declaration.

### The WBV group

The subjects in the WBV group performed the vibration exercise in a standing position. In each session, vibration was provided by a commercially available device (Galileo 2000, Novotec GmbH, Pforzheim, Germany). The subjects stood with feet side-by-side on the board, which produced lateral oscillations of the whole body. During the vibration training sessions, the subjects were barefoot to eliminate any damping of the vibration caused by footwear. The angle of flexion of the knees during the vibration exercise was set at 60°. The tridimensional acceleration was monitored by a triaxial accelerometer (TSD109F, triaxial accelerometer 5G, Biopac Systems, USA) attached to the skin at the level of the lumbar spine (L3) and normalised by body weight (g).

During the first 2 weeks of training, the WBV group performed three sets of 1 minute vibration with a frequency of 12.6 Hz of vibration stimulus, separated by 1 minute resting periods. The training load was increased systematically during the following 6 weeks, increasing by one set each week until the 6 sets of WBV that we consider to be the load of this intervention. The resting period between sets was 1 minute. The vertical amplitude of WBV was set at 3 mm. The duration of the WBV programme was about 30 minutes, which included 10 minutes warm-up consisting of 5 minutes of bicycling at 50 W and 5 minutes of static stretching for the quadriceps and triceps surae muscle.

### The Walking group

The Walking group trained outdoors. Each 1-hour session of walking was interspaced with two periods of 5 minutes each that included stretching exercises. Two research assistants, who were experienced physical education graduates, supervised this group.

### BMD assessment

At baseline and at 8 months, BMD (g·cm^-2^) of the right proximal femur (femoral neck, trochanter and Ward's triangle) and lumbar spine were assessed using dual-energy X-ray absorptiometry (DXA, Norland Excell Plus; Norland Inc., Fort Atkinson, USA).

Standard positioning was used with anterior-posterior scanning of the right proximal femur and the lumbar column. The same experienced technician performed all the scans. In our laboratory, the day-to-day precision (Coefficient of Variation %) was about 1% at lumbar and femoral neck sites, and it was about 1.2% at the rest of femoral sites.

### Balance assessment

Postural balance was assessed with a blind flamingo test, in which the barefoot subject stood on one leg, while the other leg was flexed at knee level and held at the ankle by the hand of the same side of the body, and with eyes closed. The number of trials that the subject needed to complete 30 s of the static position (the chronometer was stopped whenever the subject did not comply with the protocol conditions) was measured. The outcome was expressed as number of trials (= number of falls + 1). In our group, the test-retest intra-observer reliability coefficient of this test calculated (Intra Class Coefficient = 0.83) can be considered as acceptable for field testing in Spanish adults [22].

### Statistical analysis

Mean and standard deviation (SD) are given as descriptive statistics. Baseline characteristics were compared using Student's *t*-test for independent samples. The effects between groups were tested by ANOVA for repeated measurements, adjusted by body weight, age and baseline data. All analyses were performed with SPSS version 13.0 software. A result was considered statistically significant when the *P *value was < 0.05 for primary outcomes (BMD) and < 0.01 for secondary outcomes.

## Results

No difference in the compliance of programmes was detected, and 78% of participants completed the exercise programmes. In the WBV group, the mean frequency of attendance was 2.7 (SD 0.7) times per week, and no vibration-related side-effect or any adverse reaction was observed. In the Walking group, the mean attendance was 2.8(SD 0.8) days per week.

The baseline characteristics of both groups are given in Table 1. The groups were matched by age and weight, which are major determinant anthropometric variables on the strain in WBV training. However, the WBV group had a trend (p > .200) of higher weight and BMI than the walking group. Table 2 also shows some imbalances between the groups, so the changes were analysed by adjusting baseline data and age. After 8 months, the BMD at the femoral neck of the WBV group was increased 4.3% (*P *= 0.011) compared to the Walking group. The comparison of the changes in BMD at other sites on the hip showed a trend for the higher effectiveness of the vibratory exercise, but the difference did not reach statistical significance. In contrast, BMD at the lumbar spine was unaltered in both groups. The WBV group showed improved balance (29%), while the Walking group did not. The WBV group reduced more the BMI than the Walking group (3%; *P *= 0.049).

The lateral acceleration received by the WBV group at the lumbar spine (L3) (median 3.3 g, SD 1.3; maximal 11.6 g, SD 6.5) was greater (*P *< 0.001) than the vertical acceleration (median: 0.7 g, SD 0.5; maximal 6.4, SD 4.6).

## Discussion

### Summary of main findings

The main finding of the study was that the vibratory exercise on a reciprocating plate was more effective than walking for improving balance and BMD at the femoral neck. The adaptation of bone to physical activity and mechanical loading is crucial to the improvement and/or maintenance of bone mass and strength[18,23]. According to conventional wisdom, the stimulus should be different from what usually occurs in daily living to stimulate an adaptation of the bone tissue[24]. However, recent studies suggested that extremely low magnitude but high-frequency mechanical vibration can strongly influence bone morphology[13,25] because of the reverberation. Lodder et al. [26] calculated that with increasing age, BMD in women decreases 0.005 g/cm^2 ^per year at femoral neck (95% CI, 0.001 to 0.006 decreases) and 0.006 g/cm^2 ^per year at lumbar spine (95% CI, 0.00 to 0.007 decreases) excluding the influence of corticosteroid use. Therefore, preventing bone loss is a clinically relevant effect. The current study showed a significantly (*P *= 0.011) and clinically relevant mean effect preventing bone loss at femoral neck (0.02 g/cm^2 ^increase) but the mean effect at lumbar spine (0.01 g/cm^2 ^decrease) was not clinically relevant. More in detail, seven participants in the vibratory group and three in the walking group prevented bone mass density loss (no change or improvement in BMD) at femoral neck. A similar trend of the number of preventions was observed at the lumbar spine, but the mean of improvements were lower. In a whole, walking program did not prevent bone loss. The other positive finding of the current vibrating training programme was the high frequency of attendance at sessions (90%) of the participants who completed the programme. The profile of the sample (highly sedentary postmenopausal women) showing retention of the current programmes (78%) is similar but slightly lower than that of previous community group-based strategies to promote exercise in the elderly population (80–90%)[27]. However, strategies to improve retention of the programme should be pursued (musical environment, behavioural education, etc.).

### Comparison with the literature

#### Bone mass density

Although moderate-intensity aerobic exercise interventions usually documented positive, but not statistically significant, increases in bone mass[28]; Verschueren et al. [15] reported positive effects on hip BMD but not in total body or lumbar spine BMD after 6 months of WBV using an up-and-down plate, lower amplitudes (1.7–2.5 mm) and higher frequencies (35–40 Hz) than the current study. However, these results reflected a similar trend of adaptation to the current study. Russo et al[20] did not find any improvement in bone characteristics after 6 months of WBV training with a reciprocating plate with two sessions per week. Therefore, the number of sessions per week seems to play an important role to obtain the desired effect.

Torvinen et al[3] reported no effect on bones of healthy young adults after 8 months of vertical WBV with 3–5 sessions per week using a lower amplitude (2 mm), different frequencies (15–45 Hz) and a multidirectional vibration exposure of the body by variation of body position on the plate. The variability of the program contributed to make standing on the platform less monotonous, but distributed the mechanical strains in different body sites. Therefore, the lack of effects on bone in the study reported by Torvinen et al[3] could be explained partially because the study used lower vertical amplitudes than the current study, which are associated to a less mechanical impact, and the number of mechanical strains were shared by more anatomical sites. In addition, the sample population was younger than that of the current study.

On the other hand, the non-significance at the lumbar level can be attributed to the partial knee flexion during the vibratory exercise reducing the effects of the mechanical impact [3]. Nevertheless, Rubin et al[18] found greater strain in the vertical axis than in the lateral axis using different devices based on platforms oscillating the plate up-and-down. In contrast, the current study had higher lateral than vertical acceleration by using a reciprocating plate that oscillated on a central axis so that, when half of the platform is up the other half is down, causing a continuous balance of hips.

Yamazaki et al[8] demonstrated that moderate or walking exercise in postmenopausal women with osteopenia/osteoporosis maintained lumbar BMD via a suppression of bone turnover. Several studies showed that the effect of exercise on lumbar BMD in postmenopausal women seems to be quite modest (exercise > 1% versus control < 1%)[29] or even non-existent[30], and the walking program of the current study did not induce any effect, which could be attributed, in part, to the better health status of the subjects of the current study (no case of osteopenia/osteoporosis).

#### Balance

Six-week WBV programs with the use of a reciprocating plate at 3 sessions per week could reduce the declining balance in the elderly using 4–6 sets of 30–60 s at 35–40 Hz[10] or 4 sets of 60 s at 10–26 Hz [16]. The current 8-month trial showed that a vibrating exercise with a reciprocating plate could improve balance in postmenopausal women. In contrast, studies of 8 months using an up-and-down plate did not show any improvement of balance[3]. This difference between devices could be explained, in part, because: a) the balance and the strength of lower limbs declines with age, particularly in the lateral direction[31], the direction in which the reciprocating plate used in the current study showed the greater mechanical acceleration; b) the studies differed in the methodology of balance assessment. Therefore, further research is required to determine the adequate dose-response of vibration training needed to improve balance.

#### Body mass index

The current study reported a positive effect of WBV at 12.6 Hz on BMI compared to walking group. This amelioration could be partly attributable to the trend of the higher BMI of WBV group at baseline. Other previous study did not find this positive effect (p < .05) on weight loss using WBV at higher frequencies (25–45 Hz) compared to a control group although they also found a trend of weight loss[3]. Other authors also reported that vibratory training at 35–40 Hz was more effective to reduce fat mass than resistance training [15]. In contrast, another WBV at 35–40 Hz program in untrained young females did not show a change in weight and authors reported a small but significant increase of fat free mass[32]. On the whole, further research combining the analysis of the changes of fat free mass and lean mass is needed to elucidate this controversy and the mechanisms of weight change.

### Limitations

The main limitations of the current study are the sample size, the characteristics of the sample and the type of device employed. The size of sample could limit the chance of finding a significant effect on the BMD of the lumbar spine, but the mean of change (-0.01 gr/cm^2^) and the mean effect compared to walking group (0%) lacked of clinical relevance. In addition, this lack of effect is consistent with previous studies[15]. Changes of BMD at the trochanter and Ward's triangle were close to reach statistical significance, possibly due to the small sample size because the statistical power was 30%. In addition, the observed mean effect (3–5%) was greater than the reliability of the measurements (1.2%). Therefore, we could consider that the WBV was more effective to prevent the bone mass density loss at hip area (femoral neck, Ward's Triangle and trochanter) than the walking programme.

The generalisation of results has to be largely confined to healthy postmenopausal women, because we recruited this type of population to reduce the influence of some potentially contaminant variables (hormone replacement therapy, metabolic disorders, malnutrition etc.)[21]. Rubin et al. [25] and Torvinen et al. [3] speculated that a population with osteopenia or osteoporosis could obtain greater increases of BMD due to low baseline scores. In this sense, the Walking group showed a trend of lowers BMI, BMD and balance at baseline because the randomisation procedure applied to sample was not blocked by means of these variables. The imbalance of BMD could difficult to find more positive effects of the WBV group compared to the Walking group, but the imbalance of BMI could benefit to find comparative ameliorations in WBV.

On the other hand, the results obtained has to be restricted to devices designed to produce reciprocating vertical displacements on the left and right side of a fulcrum, which increases the lateral accelerations. Nevertheless, this type of device is one of the most frequently chosen for clinical use and for sport training. In addition, because the vertical strain was lower than the lateral strain, the effects of vibratory training on the lumbar spine could require a longer period of training or extra loading (e.g. a back-pack with weights, a higher amplitude of vibration or less knee flexion to reduce the attenuation of vibration throughout the body).

### Implications and future research

Professionals could expect greater reductions of bone fracture risk by prescribing WBV exercise rather than a walking program alone. Therefore, a vibration loading with low amplitude (3 mm) and medium intensity (12.6 Hz), could be used to prevent age-related bone loss at the hip, specially in frail populations. However, knowledge of the optimal dosage of vibratory exercise requires further research. In addition, the effects of different doses of vibratory exercise and the effects of a mixed vibratory and walking exercise are unknown.

## Conclusion

The 8-month vibratory exercise is feasible and more effective than walking to improve two major determinants of bone fractures: hip BMD and balance.

## Competing interests

The author(s) declare that they have no competing interests.

## Authors' contributions

NG was involved in the conception, planning and designing this study, the acquisition of data, analysis and interpretation of data, and writing the manuscript. AR was involved in the planning and organising this research, the acquisition of data, analysis and interpretation of data, and drafting the manuscript. AL was involved in the acquisition of data and assisting in the writing of manuscript. All authors read and approved the final manuscript.

## Pre-publication history

The pre-publication history for this paper can be accessed here:



## Figures and Tables

**Table 1:** Baseline characteristics of the sample

Assessment	Vibratory Group	Walking Group	P†
N	14	14	
Age (years)	66 (6)	66 (4)	.916
Postmenopausal age (years)	11 (6)	12 (5)	.720
Weight (kg)	70 (11)	67 (7)	.516
Height (cm)	156 (4)	157 (5)	.429
Osteocalcin (ng/ml)	26.1 (5.0)	27.6 (4.9)	.424
Deoxypyridinoline (nM/mM creat)	4.9 (2.0)	6.3 (2.4)	.112

*Values expressed as mean (SD)

†P of the T Student.

**Table 2:** Comparative effects of vibratory (N = 14) and walking-based exercise (N = 14) programs in postmenopausal women.

		Baseline	Change to 8 months	Treatment effect	P†
		
		Mean (SD)	Mean (95%CI)	Mean (95%CI)	
BMD (gr·m^-2^)					
Lumbar spine	Vibratory	0.95 (0.12)	-0.01 (-0.04 to 0.02)	0.00 (-0.03 to 0.03)	.983
	Walking	0.83 (0.13)	-0.01 (-0.02 to 0.01)		
Femoral neck	Vibratory	0.79 (0.10)	0.02 (-0.01 to 0.04)	0.03 (0.01 to 0.06)	.011
	Walking	0.78 (0.13)	-0.02 (-0.03 to 0.00)		
Trochanter	Vibratory	0.68 (0.08)	0.01 (-0.01 to 0.02)	0.02 (-0.01 to 0.04)	.084
	Walking	0.60 (0.10)	-0.01 (-0.02 to 0.02)		
Ward's triangle	Vibratory	0.63 (0.11)	0.04 (-0.01 to 0.09)	0.03 (-0.02 to 0.09)	.070
	Walking	0.58 (0.12)	0.01 (-0.02 to 0.02)		
Balance (trials)	Vibratory	9.4 (5.1)	-2.7 (-5.7 to -0.1)	-3.2 (-6.3 to -0.2)	.023
	Walking	11.3 (3.9)	0.5 (-0.9 to 0.6)		
BMI (kg/m^2^)	Vibratory	28.8 (4.3)	-0.90 (-1.8 to -0.1)	-0.8 (-1.7 to 0.0)	.049
	Walking	27.3 (2.9)	-0.1 (-0.3 to 0.2)		

BMD = Bone mineral density; BMI = Body mass index.

* Values expressed as mean (SD) with the 95% confidence intervals.

† P values of ANOVA for repeated measures, adjusted by baseline data and age to compare differences between groups at 8 months
